# Specific versus Non-Specific Exercises for Chronic Neck or Shoulder Pain: A Systematic Review

**DOI:** 10.3390/jcm10245946

**Published:** 2021-12-18

**Authors:** Lirios Dueñas, Marta Aguilar-Rodríguez, Lennard Voogt, Enrique Lluch, Filip Struyf, Michel G. C. A. M. Mertens, Kayleigh De Meulemeester, Mira Meeus

**Affiliations:** 1 Department of Physiotherapy, Faculty of Physiotherapy, University of Valencia, 46010 Valencia, Spain; lirios.duenas@uv.es (L.D.); enrique.lluch@uv.es (E.L.); 2 Physiotherapy in Motion, Multi-Specialty Research Group (PTinMOTION), Department of Physiotherapy, University of Valencia, 46010 Valencia, Spain; 3 Research Centre for Health Care Innovations, Rotterdam University of Applied Sciences, 3015 GG Rotterdam, The Netherlands; l.p.voogt@hr.nl; 4 Pain in Motion Research Group (PAIN), Department of Physiotherapy, Human Physiology and Anatomy, Faculty of Physical Education & Physiotherapy, Vrije Universiteit Brussel, 1050 Brussels, Belgium; michel.mertens@uantwerpen.be (M.G.C.A.M.M.); Kayleigh.DeMeulemeester@UGent.be (K.D.M.); mira.meeus@uantwerpen.be (M.M.); 5 MOVANT Research Group, Department of Rehabilitation Sciences and Physiotherapy, Faculty of Medicine and Health Sciences, University of Antwerp, 2000 Antwerp, Belgium; filip.struyf@uantwerpen.be; 6 Department of Rehabilitation Sciences and Physiotherapy, Faculty of Medicine and Health Sciences, Ghent University, 9000 Ghent, Belgium

**Keywords:** chronic pain, musculoskeletal pain, exercise therapy, neck pain, shoulder pain, systematic review

## Abstract

The current systematic review aimed to compare the effect of injury-focused (specific) exercises versus more general (non-specific) exercises on pain in patients with chronic neck or shoulder pain. We searched PubMed, EMBASE, and Web of Science. Two reviewers screened and selected studies, extracted outcomes, assessed risk of bias, and rated the quality of evidence. A total of nine eligible studies, represented in 13 articles, were identified, with a considerable risk of bias. One article investigated the acute effect of single bouts of exercise on pain and reported an immediate pain reduction after non-specific exercise. Regarding short-term effects, seven out of the nine studies found no differences in pain between interventions, with inconsistent results among two other studies. Concerning the long-term effects, while pain reduction seems to be favored by specific exercises (two out of four articles), the best format is still unclear. Based on the acute effects, a single bout of non-specific exercise seems to be a better option for pain-relief for patients with chronic neck or shoulder pain. For short-term effects, there are no differences in pain between specific and non-specific exercises. Regarding long-term effects, specific exercises seem to be the best option. Nevertheless, more studies are warranted.

## 1. Introduction

The prevalence of neck pain has steadily increased during the past two decades [1] and is now, second to back pain, the most common musculoskeletal disorder [2,3]. Additionally, shoulder pain is responsible for approximately 16% of all musculoskeletal complaints [4], with a yearly incidence of 15 new episodes per 1.000 patients seen in primary care settings [5]. Neck and shoulder symptoms are often persistent and recurrent, with from 40% to 50% of patients reporting persistent symptoms after 6 to 12 months [6] and 14% of patients continuing care after 2 years [7].

Successfully treating patients with chronic neck or shoulder pain (CNSP) is a challenging issue for clinicians. Exercise therapy is found to be an effective treatment strategy to relieve pain and improve patient’s level of functioning in daily activities in various chronic musculoskeletal pain disorders, including chronic neck pain [8,9,10,11] and chronic shoulder pain [12,13]. However, although the evidence for exercise therapy is strong, it is still difficult to demonstrate the superiority of one exercise approach over another in chronic pain populations [14].

Exercise interventions aim to correct biomechanical disturbances, but can also be directed to specific psychological and behavioural characteristics of chronic pain problems [14]. Naugle et al. [15] summarized the neurophysiological and hypoalgesic, effects of acute bouts of exercise in healthy and chronic pain populations in a meta-analytic review. In healthy populations, the evidence suggests that different types of acute bouts of exercise decrease the perception of experimentally induced pain. However, in patients with local muscular pain (e.g., shoulder myalgia), exercising non-painful muscles (non-specific exercises (NSE)) seems to activate generalized endogenous hypoalgesia, but exercising painful muscles (specific exercises (SE)) increases pain sensitivity in both the exercising muscle and distant locations [15,16]. While healthy people present exercise-induced hypoalgesia, regardless of the type of exercise, this mechanism seems to fail in subgroups of chronic pain patients. Among these patients, a bout of exercise can even result in a hyperalgesic response, indicating that exercise therapy should be tailored to prevent symptom flares. Nevertheless, the long-term responses to exercise therapy seem to be effective for a wide variety of chronic pain diagnoses (for a review, see Kroll, 2015 [14]).

Considering this, designing an optimal, tailor-made, exercise program for a person with CNSP requires an understanding of the underlying working mechanisms of different exercise interventions [14,17]. Additionally, the differences between the acute effects of one bout of exercise and training effects (acute, short-term, and long-term effects) should be taken into account when addressing exercise for chronic pain patients. Based on the state-of-the-art, as summarized above, the question remains as to which type of exercise, specific or non-specific, is more convenient for pain relief in people with CNSP. The aim of this systematic review was to provide a constructive overview of the existing literature reporting pain experience, following specific versus non-specific exercise therapy in CNSP patients.

## 2. Materials and Methods

### 2.1. Data Sources and Searches

This systematic review is registered in the PROSPERO register of systematic reviews (registration number: CRD42020145234) and is in accordance with the PRISMA guidelines [18]. An extensive search was conducted of the online databases PubMed, Web of Science, and Embase. Databases were searched within a 2-day period, retrospective of inception, to May 2020, with a subsequent update to January 2021. The search strategy was based on the Population, Intervention, Comparison, Outcome, Study Design (PICOS) framework and was conducted to find controlled studies (S) evaluating the effect of specific exercise programs, including neck or shoulder exercises (I), on pain (O) in CNSP patients (P), compared to non-specific exercise programs (i.e., exercises that do not specifically involve the affected region) (C). Key words from these groups were combined. The construct of the search strategy is presented in Table 1.

### 2.2. Study Selection

To be included in this review, studies had to meet the following inclusion criteria: (1) the study sample consisted of human adults (>18 years) with chronic (>3 months) neck and/or shoulder pain; (2) both treatments, SE (those focused on the neck or shoulder region) and NSE (including more generic training such as aerobic exercise, general fitness training, chain-stretching, body–mind, or other generic movement-related approaches), had to be compared in the study; (3) pain was measured as an outcome (both subjectively and objectively); (4) articles had to be written in English, Spanish, French, Dutch, or German; (5) full-text articles of original research had to be available; (6) only controlled clinical trials were allowed. Exclusion criteria determined that: (1) secondary research (reviews and meta-analysis) was not allowed; and (2) widespread pathologies and other co-morbidities could not be present.

The literature search was independently conducted, and the obtained articles were screened by two of the researchers (L.D. and M.A., both PhDs and experienced in chronic populations in a clinical setting), based on title and abstract. The full-text article was retrieved if the citation was considered potentially eligible and relevant. In the second phase, each full-text article was independently evaluated by the two researchers to see whether it fulfilled the inclusion criteria. If any of the eligibility criteria were not fulfilled, then the article was excluded. In case of disagreement, a third researcher was consulted (M.M., PhD, experienced in chronic pain research).

### 2.3. Data Extraction and Analysis

Important information from each study was selected and reported in an evidence table. The evidence table was composed of the following items: (1) reference; (2) participants’ characteristics; (3) specific intervention(s); (4) non-specific intervention(s) and reference intervention if any; (5) outcome measures and timing; (6) main results. The results regarding training effects were clustered into acute, short-term, and long-term effects; for the first days of intervention, post-intervention, and after follow-up, respectively.

### 2.4. Quality Assessment and Data Synthesis

The Cochrane Collaboration’s tool for assessing risk of bias was used (http://handbook.cochrane.org/, accessed on 24 May 2020) to assess the following domains: (1) the randomization process; (2) treatment allocation; (3) blinding of participants and personnel; (4) blinding of outcome assessors; (5) completeness of the outcome data; (6) reporting of results; (7) accounting for co-interventions; (8) other sources of bias. Item 8 was specifically focused on sample size calculation. With reference to a Cochrane review, sample size was considered inadequate if there were fewer than 50 participants per group and if power analysis was not applied and reported for relevant outcome measures [19].

After clustering the results based on exercise modes and timing of assessments, the overall quality of evidence per cluster was determined by applying the Grades of Recommendation, Assessment, Development, and Evaluation (GRADE) approach [20]. For every cluster, a GRADE summary statement is provided under the respective paragraph in italics.

Risk of bias assessment and grading of evidence was performed by two authors (L.D. and M.A.) independently, who were blinded from each other’s assessment. After rating the selected articles/clusters, the results of both researchers were compared, and differences were analyzed. In case of disagreement, the reviewers assessed the article/cluster a second time to obtain a consensus. When consensus could not be reached, a third opinion was provided by the last author (M.M.).

## 3. Results

### 3.1. Search Results

**Figure 1:**
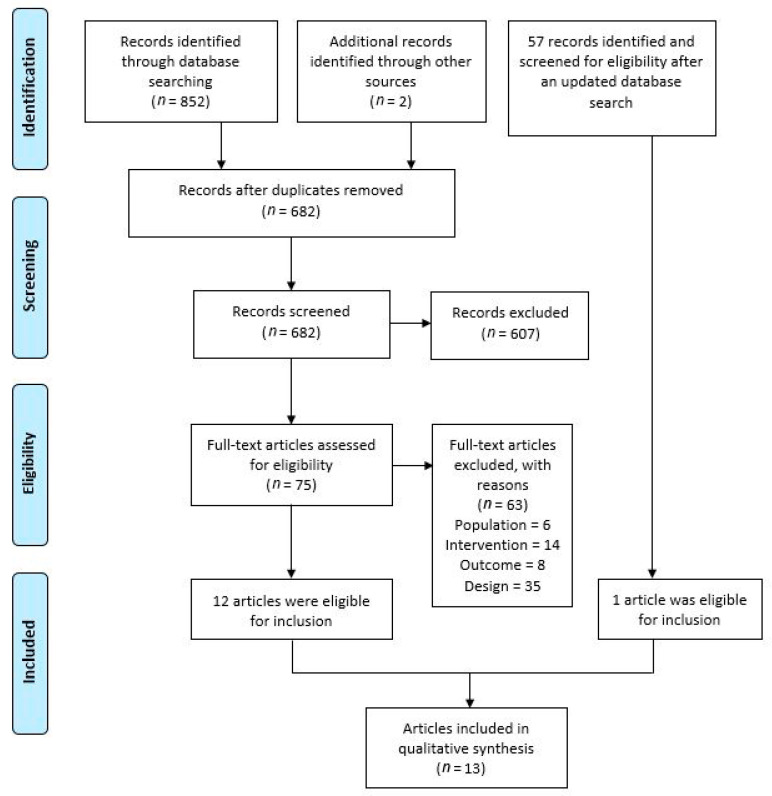
PRISMA flowchart of articles selection (adapted from Moher et al. [18]).

The initial search of all databases resulted in 852 hits. Following two consecutive screening phases on title/abstract and full text, 10 eligible records remained. After manual searching of the reference lists, two more eligible articles were identified for inclusion. A recent update identified 57 new articles, leaving one of them for inclusion in the review, after the screening phases. Thus, a total of 13 articles, reporting the results of nine different randomized controlled trials, met the inclusion criteria. The corresponding flowchart is shown in Figure 1.

### 3.2. Risk of Bias and Quality of Evidence

**Figure 2:**
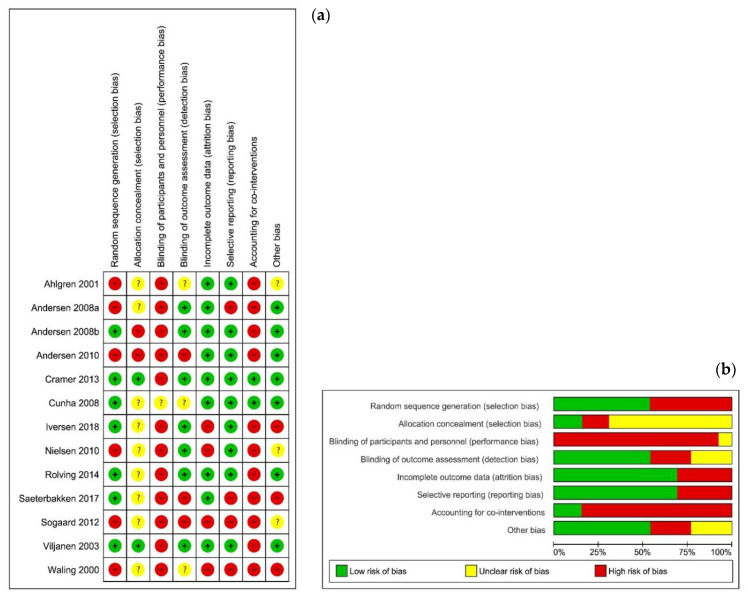
Risk of bias summary. These graphs illustrate the review authors’ judgements about each risk of bias item for each included study (**a**) and presented as percentages across all included studies (**b**). Review Manager (RevMan) 5.3. [34]. Legend: (+) indicates “low risk of bias”; (?) indicates “unclear risk of bias”; (−) indicates “high risk of bias”.

Detailed information on the individual risk of bias can be found in Figure 2. In most cases (85.6% or 89 of 104 items), the two researchers agreed. After a comparison of the 15 differences, the reviewers reached a consensus for six items. The remaining nine points of discussion were solved after a third opinion. Nine of the 13 articles provided insufficient information about the allocation concealment [21,22,23,24,25,26,27,28,29]. None of the studies reported that the therapist was blinded. Additionally, blinding of the patients was impossible, given the nature of the therapy. In one study, the patients were kept naïve for the different interventions (specific or global stretching). This study was considered as having an unclear risk of bias, because the assumptions of patients were unclear [27]. Attrition and reporting bias were mainly low. Two of the 13 articles accounted for co-interventions by recording medications and other treatments received in a diary [30] and by registering medication type and frequency [27]; the other articles did not account for co-interventions. Five articles conducted a sample size calculation [23,26,27,30,31]. Two of the 13 articles [32,33] included more than 50 subjects per group.

Information on risk of bias and the level of evidence, following the GRADE system, is presented per cluster in Table 2. Since none of the studies was double-blinded, all clusters started from a GRADE level of moderate.

### 3.3. Study Characteristics

A total of 13 articles were reviewed, originating from nine data files (from now on referred to as studies). Although one study generally generated a single article, the results of three studies generated seven articles, whose differentiating aspects can be broadly disaggregated and conveyed as follows: (a) Andersen et al. [32,33], 2008 [32] referred to the short-term effects post-intervention with pain intensity as an outcome, while 2010 [33] referred to a higher sample size and pain regions as an additional outcome. (b) Andersen et al. [26], Nielsen et al. [25] and Søgaard et al. [24] varied their timeframes and outcomes. Andersen et al. [26] assessed the short-term effects post-intervention, similar to their counterparts, but included assessments halfway through the training period and after 10-week follow-up. An analysis was completed, looking at the acute effects after one session. Pain intensity was the outcome. Nielsen et al. [25] analyzed pressure–pain thresholds (PPTs), and Søgaard et al. [24] included repetitive and stressful work tasks as a test to evaluate the training effects on pain intensity. (c) Both Ahlgren et al. [28] and Waling et al. [29] evaluated the short-term effects post-intervention, varying in their assessed pain-related outcomes (pain intensity and PPTs and pain distribution, respectively).

The number of patients in each study varied from 33 to 616. Eight out of 13 articles only included women [22,24,25,26,27,28,29,31], whereas the other articles included both men and women. A total of 1229 women and 271 men were evaluated, with a mean age varying between 37.6 ± 6.1 years [28,29] and 50.3 ± 14.8 years [22] for the women and between 39.6 ± 9.2 years [23] and 49.0 ± 1.4 years for the men [32]. Most of the patients were office workers [22,24,25,26,31,32,33] and assembly line workers [24,26]. Six out of the 13 articles did not specify the patients jobs [21,23,27,28,29,30].

Out of 1500 patients, the vast majority (a total of 1269 patients) were diagnosed with non-specific chronic neck-shoulder pain [21,22,23,27,30,31,32,33]. The remaining 231 patients were diagnosed with trapezius myalgia [24,25,26,28,29]. No study analyzed patients with shoulder pain as a standalone disorder, and all were part of a sample of neck–shoulder pain patients.

Concerning the SE, strengthening exercises using dumbbells were used in six of the articles [24,25,26,31,32,33], followed by air machines [28,29], and elastic band [21,22,23] or isometric exercises using a towel [30]. One study included conventional auto-passive stretching as specific exercise [27].

The NSEs included in the studies were bicycle ergometer training [24,25,26], nordic walking [22], advice about staying physically active [21,23,32,33], global stretching [27], and body–mind therapies such as yoga [30], relaxation [21,31] or body awareness [21,28,29].

Most outcome measures concerned self-report pain measures. Visual analogue scales [22,24,26,27,28,29,30,31], 0–9 scales [32,33], and numeric rating scales [21,23] were used to evaluate pain. PPTs were measured in four of the included articles [21,25,29,30]. The other outcomes registered in the different articles were the body pain scale of the 36-item Short Form Health Survey (SF-36) [27,30], neck pain regions (n) [21,33], and pain drawings [21,29].

Frequency of therapies varied from 1 [33] to 5 times/week [30], with 3 times/week the most frequently used [21,23,24,25,26,28,29,31,32,33].

The total duration of the exercise program lasted from 6 weeks [27] to one year [32,33], with a modus of 9 to 10 weeks [22,24,25,26,28,29,30]. Follow-up varied between 6 weeks [27] and 9 months [31] after treatment ending.

All studies analyzed the short-term effects of exercise on pain. Four studies analyzed the long-term effects [22,26,27,31], and one study considered the acute effects after one exercise session [26].

Individual study results were clustered based on treatment types and follow-up effects: acute effects after one exercise session and training effects (acute, short-term, and long-term effects), as presented in Table 3, Table 4 and Table 5**.**

### 3.4. Data Synthesis

#### 3.4.1. Specific Strength vs. Non-Specific Aerobic Exercises

A total of 8 out of the 13 articles analyzed the effects of specific strength training compared to general aerobic exercises (Table 3).

- Acute effects

One article analyzed the acute effects of a single bout of exercise [26]: non-specific exercise, based on a generic aerobic program, caused an immediate post-exercise pain reduction. Specific strength training showed an immediate post-exercise pain increase during the first half of the training period that flattened near the end of the 10-week training program. Both pain increases and reductions leveled off 2 h after exercise.

- Short-term effects

Seven out of the 13 articles analyzed the short-term effects of physical exercise on pain behavior [21,22,23,24,25,26,32,33]. Both specific strength training and non-specific physical exercise programs of 10–12 weeks (20–30 min training, 2–3 days/week) resulted in a decrease in general pain [22,23,32,33], in pain during a repetitive task [24], and in the number of pain regions [33] compared to a reference intervention. However, in two articles, specific strength training for 20 min/day, 3 times/week was superior in reducing pain in general [26], pain at worst [26], and pain at rest [24] after 10 weeks of treatment in women with trapezius myalgia.

Two articles reported the effects of exercise on PPTs, reporting no differences between specific and non-specific training [21,25]. While Iversen et al. [21] found no changes after the exercise program, Nielsen et al. [25] reported that pain sensitivity at a pain-free reference muscle was decreased (i.e., higher PPTs) in response to both specific strength training (concentric and eccentric contractions) and non-specific fitness training (bicycle ergometer) after 10 weeks of exercise (20-min training, 3 days/week) in women with trapezius myalgia.

- Long-term effects

Two articles analyzed the effects of specific strength training vs. aerobic exercise 10 weeks after finishing the exercise program, with inconsistent results. Saeterbakken et al. [22] found that both exercise types (specific and non-specific) had a similar effect on pain reduction that lasted during follow-up compared with no effect in a reference group. Nevertheless, the study performed by Andersen et al. [26] reported that specific strength training resulted in significant pain reduction, in contrast to the non-specific aerobic exercise group, which consolidated in the further 10-week follow-up period.

In conclusion, there is low evidence that specific strengthening exercises and non-specific fitness training produce similar short-term effects regarding pain relief. There is only preliminary evidence that immediate acute response to exercise is more favorable for the non-specific exercise program. There is also very low evidence that the long-term effects are favored by specific strengthening exercises.

#### 3.4.2. Specific Strength vs. Body Mind Exercises

A total of 4 out of the 13 articles analyzed the effects of specific strength training compared to body mind therapies (Table 4) [28,29,30,31].

- Short-term effects

All the mentioned articles reported positive short-term effects of exercise programs on pain behavior. There were no differences between specific strength training and NSE (body–mind exercises through body awareness and yoga), as both resulted in a decrease in the intensities of pain at motion [30], pain at present, pain at worst, and pain in general [28,29] after 9 or more weeks of treatment (3 days/week).

However, there were inconsistent results in two of the articles regarding three outcomes: specific strength training during 60 min/day, 3 times/week, reduced pain at worst after 10 weeks of treatment in women with trapezius myalgia compared to body-awareness exercises [28]. Yoga classes for 90 min/week reduced pain in general and bodily pain items from SF-36, after 9 weeks of practice, in patients with non-specific neck pain, compared to specific strength exercises [30].

Two articles reported the effects of exercise on PPTs [29,30]. Cramer et al. [30] demonstrated better results with non-specific interventions: yoga exercises, practiced with an instructor for 90 min/week for 9 weeks, decreased pressure sensitivity in non-specific neck pain patients compared to strength training for 10 min/day. The study of Waling et al. [29] found no differences between SE and NSE: pressure sensitivity significantly decreased at four myofascial trigger points of the trapezius muscle in both exercise regimens, compared to a reference group.

For pain drawings, no changes were seen in the extent of painful body area in any of the exercise groups (body awareness and specific strength) [29].

- Long-term effects

One article analyzed the effects of a 13-week specific-strength training (3 days/week) compared to relaxation training and a reference group, after 9 months follow-up, concluding that no difference was found in neck pain intensity in questions concerning neck pain disability between the three groups in a sample of 393 female office workers with chronic non-specific neck pain [31].

There is moderate evidence that exercises reduce pain in the short-term compared to a reference group and there seems to be no difference for different types of exercise. For the long-term, there is only preliminary evidence that there is no difference between exercise groups and the reference group.

#### 3.4.3. Specific Stretch vs. General Stretch Exercises

- Short and long-term effects

One article investigated the effects, both short and long-term, of specific versus general stretches on pain reduction (Table 5) [27]. These authors suggest that conventional specific stretching and muscle chain stretching (30 min, 2 times/week), in association with manual therapy (30 min, 2 times/week), were equally effective in reducing the pain of female patients with chronic neck pain, both post-treatment and at six weeks after ending the treatment.

There is preliminary evidence that specific and non-specific stretching exercises are equally beneficial for pain reduction in female patients with chronic neck pain, although more studies are needed.

## 4. Discussion

This is the first systematic review specifically examining the effect of SE compared with NSE on pain in the rehabilitation of patients with CNSP.

The aim of this review was to evaluate the effect of SE, involving exercises focused on the neck and/or shoulder region, focused on CNSP patients, looking for pain reduction/increases compared to NSE.

There is considerable evidence of pain reduction after an exercise program, both specific and non-specific, in the short- and long-term [22,24,26,28,29,32,33]. For the short-term effects, 9 out of 13 articles did not favor a particular type of exercise [21,22,23,27,28,29,31,32,33], while 3 articles [24,25,26] found better effects on pain for specific training, and the other article favored non-specific training [30]. With regard to the long-term effects of exercise on pain, 3 out of 4 articles found that specific [26], or both exercise types independently (specific and non-specific) [22,27], had a lasting effect on pain reduction. The other article found that exercise had no long-term effects on pain [31]. Nevertheless, regarding the acute effect of single bouts of exercise, only one article assessed this aspect [26], reporting an immediate pain reduction after non-specific exercise in contrast to specific resistance exercises. Consequently, more research is needed, specifically about acute and long-term effects.

These results are in line with a Cochrane systematic review evaluating the use of motor control as a specific exercise strategy among a chronic non-specific neck pain population [8]. The study suggested that specific motor control exercises were not superior to more general exercise strategies. Furthermore, the review of Booth et al. [35] did not provide evidence for the superiority of one exercise type in chronic musculoskeletal pain conditions. Therefore, the type of exercise might be less important than the act of doing exercise. Sluka et al. [36] suggest that this lack of specificity of exercise type may be related to the multiple and widespread mechanisms by which exercise works to reduce pain.

Although this aspect is out of the scope of the present review, it is interesting to try to elucidate the mechanisms that could explain our findings. The reason that the research to date has not shown any specific exercise to be superior may be that psychological and/or neurophysiological factors that are common to all exercise approaches have the greatest mediating effects on pain [37]. If changes in pain and disability occur without changes in physical function, then specific modalities of exercise and their dosage seem to be less relevant in chronic musculoskeletal pain [35,38]. It is tempting to speculate that exercise can indeed desensitize the central nervous system. This hypothesis has recently been supported through a review of the current evidence on the central mechanisms underlying exercise-induced pain and analgesia [39].

Exercise is likely to be most effective if tailored to individual patients with spinal pain. As Falla and Hodges [40] stated, current exercise programs for spinal pain treatment often rely on a one-size-fits-all approach and usually fall short of success. These authors provide evidence supporting the hypothesis that the outcome of exercise interventions can be optimized when targeted to the *right* people and adapted to the individual’s presentation. In the same line, tailoring exercise to individual patients has been recommended for chronic musculoskeletal pain [17,41], which requires an initial assessment to understand the biological, psychological, and social factors contributing to pain and disability [35]. The dominant pain mechanism must also be considered to optimize exercise prescription. Indeed, a recent systematic review concluded that global (non-specific) exercises are preferred in nociplastic pain conditions, while more SE should be emphasized in non-nociplastic conditions [42]. In the present review, however, all the included studies used standardized exercise programs and no prior assessment was made to determine the patient’s profile.

The level of supervision is also an important aspect in promoting treatment adherence and patients’ motivation [35]. Supervised exercise programs have been recommended for chronic musculoskeletal pain [17,43]. In the present review, all studies but one [23] included supervised exercise sessions. This could be the reason that the drop-out rate was relatively low in those studies.

Finally, ongoing self-monitoring can be helpful to identify barriers to [14] and facilitators of exercise participation, motivate positive exercise behavior and increase participation [44]. In the present review, only five articles out of 13 did not use diaries to register their adherence to the exercise programs [22,24,25,26,31], which could also explain the high participation rate.

Thus, supervised exercise, individualized therapy, and self-management techniques may help to promote a successful rehabilitation program [14]; however, the quality of trials assessing these interventions is low [43], and further research is warranted.

### Limitations

First, the main weakness of this review is the risk of bias. Random sequence generation, accounted co-interventions, and concealment of allocation were often not attained. Therefore, a note of caution is due here. Most studies failed to achieve blinding of the patients. Furthermore, the majority of studies relied on self-reported measures, prohibiting blinding of the assessors as well. Although blinding participants and therapists in an exercise trial is difficult to implement and cannot obviate the risk of bias, future studies should endeavor to limit the potential bias with the appropriate blinding of at least the assessors. Keeping the patients and therapists naïve regarding the received treatment should be attempted, as specific expectations and beliefs could influence outcomes. Assuming naïve patients is only possible in the studies evaluating different exercise modalities. This should be considered in future studies. Second, the number of RCTs included was low. The limited number of studies published in this area also raises the possibility of publication bias. Third, patient activity between post-test and follow-up was not controlled in any study. Finally, only four articles analyzed the follow-up period [22,26,27,31]. In two of these, the follow-up was limited to less than three months, which seems to be insufficient, as CNSP can last for up to several years [26,27]. This aspect limits any comment on the maintenance of the effects of exercise. Ongoing research including acute and follow-up schemes over six months is required to further validate our findings and determine the long-term effects of the intervention.

Furthermore, there was a lack of uniformity in the obtained results regarding the differences in the benefits between specific and non-specific exercises. The term “specific exercise” has been used to describe different types of exercises, such as stabilization [45], strengthening [46], individualized [47], supervised [48], and even what appear to be general exercises [49]. Non-specific exercise protocols usually address general flexibility, strength, and/or endurance training, including all body regions. Such inconsistency, together with an incomplete description of exercise details regarding dosage [8], are a possible reason for the inconsistent results found in different chronic pain populations. Therefore, the working mechanisms and exact definition and dosage of the exercise therapy modalities need to be further elaborated.

## 5. Conclusions

This systematic review shows interesting findings for pain relief with regard to training effects using specific and/or non-specific exercise for CNSP. Both specific (neck and/or shoulder exercises) and NSE seem to be effective for short-term pain reduction in patients with CNSP.

Based on the acute effects, there is only preliminary evidence that a bout of non-specific exercise seems to be more tolerable for patients with CNSP, overcoming the exacerbation in the beginning. Regarding the long-term effects, SE seems to be the best option, although the evidence for this is very limited. As the evidence is still rather restricted, this review highlights the need for further RCTs comparing the effects of injury-focused (specific) exercises versus more general (non-specific) exercises, and a need to better understand the definition and dosage of exercise therapy modalities to improve clinical application.

## Figures and Tables

**Table 1:** Search strategy.

	Keywords
Group 1 (Population)	“Arthralgia”(MeSH) OR “Bursitis”(MeSH) OR “Cervical vertebrae”(MeSH) OR “Chronic pain”(MeSH) OR “Hernia”(MeSH) OR “Intervertebral Disc Displacement”(MeSH) OR “Musculoskeletal System”(MeSH) OR “Myalgia”(MeSH) OR “Myofascial Pain Syndromes”(MeSH) OR “Neck”(MeSH) OR “Neck Pain”(MeSH) OR “Osteoarthritis”(MeSH) OR “Pain, intractable”(MeSH) OR “Rotator cuff”(MeSH) OR “Shoulder Impingement Syndrome”(MeSH) OR “Shoulder Pain”(MeSH) OR “Shoulder”(MeSH) OR “Tendinopathy”(MeSH) OR “Whiplash Injuries”(MeSH) OR (Chronic pain OR Intractable pain OR Joint Pain OR Muscle Pain OR Musculoskeletal pain OR Myalgia OR Myofascial pain OR Osteoarthritis OR Persistent pain OR Severe pain OR Tendinopathy) AND (Neck OR Shoulder OR Cervical OR Adhesive capsulitis OR Frozen shoulder OR Impingement OR Rotator cuff OR Spinal disc herniation OR Spinal pain OR Whiplash)
Group 2 (Intervention)	“Exercise”(MeSH) OR “Exercise Therapy”(MeSH) OR “Cervical Vertebrae”(MeSH) OR “Functional Laterality”(MeSH) OR “Isometric Contraction”(MeSH) OR “Isotonic Contraction”(MeSH) OR “Muscle Strength”(MeSH) OR “Muscle Stretching Exercises”(MeSH) OR “Neck”(MeSH) OR “Plyometric Exercise”(MeSH) OR “Proprioception”(MeSH) OR “Resistance Training”(MeSH) OR “Shoulder”(MeSH) OR “Visual Motor Coordination”(MeSH) OR “Weight Lifting”(MeSH) OR “Weight-Bearing Exercise Program”(MeSH) OR Exercise AND (Shoulder OR Cervical OR Neck OR Abduction OR Adduction OR Balls OR Bands OR Concentric OR Coordination OR Dynamic OR Eccentric OR Extension OR External Rotation OR Flexibility OR Flexion OR Free weights OR Internal rotation OR Isometric OR Isotonic OR Kettlebell OR Motor control OR Plyometric OR Proprioception OR Red cord OR Resistance training OR Resisted OR Static OR Strength OR Strength training equipment OR Stretching OR Thera-band OR Weight-bearing exercise program OR Weights)
Group 3 (Comparison)	“Exercise”(MeSH) OR “Exercise Movement Techniques”(MeSH) OR “Exercise Therapy”(MeSH) OR “Bicycling”(MeSH) OR “Dancing”(MeSH) OR “Hydrotherapy”(MeSH) OR “Jogging”(MeSH) OR “Muscle Stretching Exercises”(MeSH) OR “Physical Fitness”(MeSH) OR “Physical Endurance”(MeSH) OR “Resistance Training”(MeSH) OR “Running”(MeSH) OR “Swimming”(MeSH) OR “Walking”(MeSH) OR “Yoga”(MeSH) OR Exercise AND (Non-specific exercise OR Non-specific training OR Aspecific OR Activity program OR Aerobic OR Alexander technique OR Aquatic exercise OR Bicycling OR Cycling OR Dancing OR Endurance OR Fitness OR General exercise OR Generic exercise OR Hydrotherapy OR Jogging OR Physical activity OR Resistance training OR Rowing OR Running OR Stretching OR Swimming OR Tai chi OR Training OR Walking OR Yoga)
Group 4 (Outcome)	“Pain”(MeSH) OR “Pain Measurement”(MeSH) OR “Analgesia”(MeSH) OR “Central Nervous System Sensitization”(MeSH) OR “Hyperalgesia”(MeSH) OR “Hypersensitivity”(MeSH) OR “Nociceptors”(MeSH) OR “Pain Management”(MeSH) OR “Pain Threshold”(MeSH) OR “Pain Perception”(MeSH) OR “Pain, Intractable”(MeSH) OR “Pain, Referred”(MeSH) OR “Somatosensory Disorders”(MeSH) OR “Visual Analogue Scale”(MeSH) OR Pain OR Pain measurement OR Algometry OR Analgesia OR Central nervous system sensitization OR Centrally mediated pain modulation OR Conditioned pain modulation OR Endogenous pain inhibition OR Endogenous pain-inhibitory mechanisms OR Exercise-induced hgperalgesia OR Hyperalgesia OR Hypersensitivity OR Hypoalgesia OR McGill OR Nociceptors OR Pain control OR Pain threshold OR Pain-relief OR Persistent pain OR Pressure pain thresholds OR Quantitative sensory testing OR Referred pain OR Sensitivity OR Somatosensory disorders OR Temporal summation OR Visual analogue scale OR Wind-up effect
Group 5 (Study design)	“Controlled Clinical Trials”(MeSH) OR Controlled clinical trials

Abbreviations: MeSH, Medical Subject Headings.

**Table 2:** Risk of bias and grading the evidence per clusters based on exercise type and exercise effects over time OR follow-up (acute, short- and long-term effects).

**1-. SPECIFIC STRENGTH VS. NON-SPECIFIC AEROBIC**					
**1.1. ACUTE EFFECTS**					
**Study**	**VAS/NRS**			**Risk of bias**	**GRADE**
	**SI**	**NSI**	**REF**		
Andersen et al. [26]	↑ VAS in untrained patients = VAS in trained patients	↓ VAS	ø	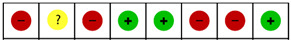	⊕⊖⊖⊖
**1.2. SHORT-TERM EFFECTS**					
Andersen et al. [32]	= pain (0–9 scale) in neck and shoulder pain patients		= pain (0–9 scale) in neck and shoulder pain patients	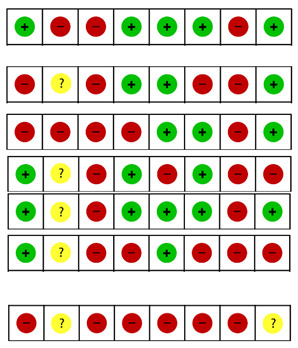	⊕⊕⊖⊖
Andersen et al. [26]	VAS in general ↓ VAS at worst ↓	VAS in general = VAS at worst =			
Andersen et al. [33]	↓ pain (0–9 scale) > than REF		↓ pain (0–9 scale)		
Iversen et al. [21]	NRS =		ø		
Rolving et al. [23]	↓ NRS		ø		
Saeterbakken et al. [22]	VAS intensity ↓		VAS intensity =		
Søgaard et al. [24]	VAS at rest ↓ > than NSI and REF VAS during repetitive and stress tasks =	VAS at rest = VAS during repetitive tasks ↓ > than SI and REF	VAS at rest and during repetitive and stress tasks =		
**1.3. LONG-TERM EFFECTS**					
Andersen et al. [26]	VAS in general ↓ VAS at worst ↓ and keeps on, from short-term effects, stable and < than NSI and REF (therapy effects remained)	VAS in general = VAS at worst = ø		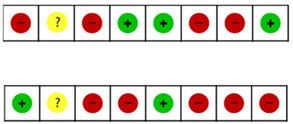	⊕⊖⊖⊖
Saeterbakken et al. [22]	VAS intensity ↓		VAS intensity =		
**2-. SPECIFIC STRENGTH VS. BODY-MIND**					
**2.1.ACUTE EFFECTS:** no study					
**2.2.** **SHORT-TERM EFFECTS**					
Ahlgren et al. [28]	Overall VAS ↓ ↓ VAS at worst > than REF	Overall VAS ↓	Overall VAS =	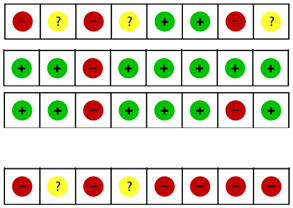	⊕⊕⊕⊖
Cramer et al. [30]	VAS at motion ↓ VAS intensity =	VAS at motion ↓ VAS intensity ↓ > than SI	ø		
Viljanen et al. [31]	= VAS				
Waling et al. [29]	Pain at present = Pain in general = VAS at worst ↓ > than REF	Overall pain =	Overall pain =		
	VAS at present and VAS at worst “exercisers” ↓ > than REF				
**2.3.** **LONG -TERM EFFECTS**					
Viljanen et al. [31]	= VAS			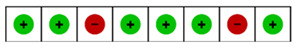	⊕⊖⊖⊖
**3-. SPECIFIC STRETCH VS. GENERAL STRETCH**					
**3.1.ACUTE EFFECTS:** no study					
**3.2.** **SHORT-TERM EFFECTS**					
Cunha et al. [27]	↓ VAS		ø	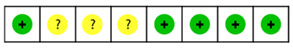	⊕⊖⊖⊖
**3.3. LONG -TERM EFFECTS**					
Cunha et al. [27]	↓ VAS (from baseline to 6 w follow-up post-intervention)		ø	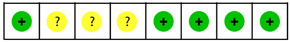	⊕⊖⊖⊖

Abbreviations: NRS, numerical rating scale; NSI, non-specific intervention; REF, reference group; SI, specific intervention; VAS, visual analogical scale; W, week/s. Legend: ↑ indicates “increased/higher”; ↓ indicates “decreased/lower”; = indicates “no change”; (+) indicates “low risk of bias”; (?) indicates “unclear risk of bias”; (−) indicates “high risk of bias”. ø: not evaluated.

**Table 3:** Study characteristics. Specific strength vs. non-specific aerobic exercises.

Reference	Characteristics of Participants	Specific Intervention (SI)	Non-Specific Intervention (NSI) + Reference Intervention (REF)	Outcome Measures / Follow-Up Period	Main Results
Andersen et al. [32]	Office workers with neck or shoulder pain > 3/9 and ≥3 m Neck: *n* = 182, ♀ (44 ± 0.9 y); ♂ (49 ± 1.4 y) Shoulder: *n* = 94, ♀ (44 ± 1.1 y); ♂ (48 ± 1.4 y) **SI group** Neck: *n* = 61 Shoulder: *n* = 41 **NSI group** Neck: *n* = 59 Shoulder: *n* = 46 **REF group** Neck: *n* = 62 Shoulder: *n* = 37 ITT analysis	**Specific neck-shoulder dynamic and static strengthening ex with dumbbells and inelastic strap** - 20′ × 3/w for 1 y - 2/3 supervised - during working hours - Load ↑ when they performed > 15 reps/ex - Last 15″: high-speed dynamic power ex (kayaking or ergometer rowing)	**General fitness training** - 1 h/w during working hours for 1 y - Filled in a “contract”, writing the ways to include + physical act in their lives - Swimming, fitness clubs, all-round strength and aerobic fitness lessons (1–4 visits/m), walking group sessions (step counters), group sessions of Nordic walking, aerobic fitness, etc. **REF group** - No physical act - Workplace ergonomics, stress management, etc. - =supervision as SI and NSI	**Pain:** - Pain intensity during last 3 m (0–9 scale) ~ baseline ~ post-intervention (1 y)	**Short-term effects (post-intervention):** PAIN INTENSITY: - = SI, NSI and REF
Andersen et al. [33]	Same characteristics as Andersen et al. [32] *n* (at baseline) = 616 397 ♀ (44.6 y); 219 ♂ (45.7 y) (67 excluded/withdrew = 549) **SI group** *n* = 180 **NSI group** *n* = 187 **REF group** *n* = 182 ITT analysis			**Pain:** - Pain intensity during last 3 m (0–9 scale) - Pain regions (*n* = 0–11), with a VAS ≥ 3/9 ~ baseline ~ post-intervention (1 y)	**Short-term effects (post-intervention):** PAIN INTENSITY: - ↓ SI, NSI and REF - ΔSI and ΔNSI > ΔREF Pain regions (*n*): - ↓ SI and NSI; =REF
Andersen et al. [26]	♀ office workers (30–60 y), assembly line or office workers, with CNSP (≥30 d in the last y), reporting pain ≥ 1 episode/w + pain intensities at T0 ≥ 3 (0–9 scale) + diagnosed as trapezius myalgia. *n* = 48 ♀ (end 43 ♀) 44 ± 8 y **SI group** *n* = 18 ♀. 44 ± 8 y **NSI group** *n* = 16 ♀. 49 ± 7 y **REF group** *n* = 14 ♀ (end 9 ♀) 48 ± 11 y	**5 neck–shoulder specific strengthening ex with dumbbells** - 20′ × 3/w supervised for 10 w - 3 sets (25–35″)/ex - High intensity (consecutive concentric and eccentric muscle contractions without pause or breaks) - Load progressively ↑ 12 → 8 RM; (~70 → 80% max intensity)	**Bicycle ergometer training** - 20′ × 3/w supervised for 10 w - High intensity - Intensity progressively ↑ 50 → 70% (Vo_2max_) **REF group** - 1 h/w for 10 w - No physical act - Health counseling on group + on individual level (workplace ergonomics, diet, relaxation, ...) - = supervision as SI and NSI	**Pain** (diary report): - General pain (VAS1) - Pain at worst (VAS2) - Pain immediately before the session (VAS3) - Pain immediately after the session (VAS4) ~ baseline ~ half of training period ~ post-intervention (10 w) ~ 10 w follow-up	**Acute effects after 1 ex session** **• 1st half of training period:** - VAS4-VAS3: ↑ SI; ↓ NSI. Effects lasted for 2 h - ΔNSI > ΔSI and REF • **2nd half of training period:** VAS4-VAS3: - ↓ NSI; = SI. Effects lasted for 2 h - ΔNSI > ΔSI and REF **Short-term effects (post-intervention):** VAS1,2: - ↓ SI; = NSI and REF - ΔSI > ΔNSI and REF **Long-term effects (follow-up):** VAS1,2: - ↓ SI; = NSI and REF - ΔSI > ΔNSI and REF
Nielsen et al. [25]				**PPTs:** - Painful trapezius (PPT1) - Non-painful tibialis anterior (PPT2) ~ baseline ~ post-intervention (10 w)	**Short-term effects (post-intervention):** PPT1: - ↑ SI; = NSI and REF - Δmyalgia < Δcontrols PPT2: - ↑ SI and NSI; =REF - Δmyalgia < Δcontrols
Søgaard et al. [24]	♀ performing monotonous & repetitive work tasks + trapezius myalgia (30–60 y) *n* = 47 ♀ (end 39 ♀) **SI group** *n* = 16 ♀ 44.6 ± 8.5 y **NSI group** *n* = 15 ♀ 45.5 ± 8.0 y **REF group** *n* = 16 ♀ (end 8 ♀) 42.5 ± 11.1 y			**Pain:** - At rest (VAS1): measured prior to the repetitive task - During repetitive task (VAS2): pegboard work (40′). VAS every 5′. Changes in VAS slope (time curve) (mm/min)- VAS after 120′ rest immediately before a stressful Stroop task (VAS3). - VAS immediately after the stressful Stroop task (VAS4) ~ baseline (2 d before the intervention) ~ post-intervention (10 w)	**Short-term effects (post-intervention):** VAS 1: - ↓ SI; = NSI and REF - ΔSI >ΔNSI = ΔREF VAS2: - ↓ NSI; = SI & REF - ΔNSI > ΔSI = ΔREF VAS3, 4: -ΔSI = ΔNSI = ΔREF
Iversen et al. [21]	Patients (16–70 y) + non-specific neck pain ≥ 3 m or ≥ 2 times ≥ 4 w in the past y and worst neck pain in last 2 w NRS ≥ 4 *n* = 59 (39 ♀ & 20 ♂) (end 31) **SI group** *n* = 29 (end 15) 20 ♀ & 9 ♂ 44.6 ± 8.1 y **NSI group** *n* = 30 (end 16) 19 ♀ & 11 ♂ 48.2 ± 10.6 y	**MDR + 8 neck-shoulder specific strengthening ex. with elastic bands** - 3 w MDR: patient education, stress management, group discussions - 9 w SI program: 3 times/w. Supervised at 1st and 3rd w - +1 session NSI - +3 group booster sessions - Yellow–gold Theraband^®^ - Reps/ex until muscular failure - Load progressively ↑: sets/reps/band color - Diary for daily registration	**MDR + General fitness training** - 3 w MDR: patient education, stress management, group discussions - 9 w NSI program: 1st and 3rd w, 4 and 3 supervised sessions - Introduction to group-based and individual act (circle-training, endurance, low-intensity resistance, stretching) - + 3 group booster sessions - Diary for daily registration	**Pain:** - Current neck pain (NRS1) - Pain at worst last 2 w (NRS2) - Pain at worst in last 4 w (NRS3) **PD:** Additional pain sites (*n*) **PPTs:** tibialis anterior muscle ~ baseline ~ post-intervention (12 w)	**Short-term effects (post-intervention):** NRS, pain sites and PPTs: - = SI and NSI - ΔSI = ΔNSI
Rolving et al. [23]	Patients on sick leave from work (4–16 w prior to study) due to non-specific neck pain (18–60 y) *n* = 83 (60 ♀ & 23 ♂) (end 71) **SI group** *n* = 43 (end 34) 27 ♀ & 16 ♂ 39.6 ± 9.2 y **NSI group** *n* = 40 (end 37) 33 ♀ & 7 ♂ 39.0 ± 11.0 y ITT analysis	**General fitness training and 4 specific neck–shoulder-strengthening ex. With elastic band** - 15–20′ supervised training ≥ 3 times/w for 12 w - Participants instructed to be physically active ≥ 30′/d, 3–4 h/w - 3 × 5 reps/ex - Load progressively ↑/2 w - Diary for daily registration	**General fitness training** - Participants instructed to be physically active ≥ 30′/d, 3–4 h/w for 12 w - Minimal supervision - Diary for daily registration	**Pain:** - Pain intensity during last w (0–10 scale) (NRS) ~ baseline ~ post-intervention (12 w)	**Short-term effects (post-intervention):** NRS: - ↓ SI and NSI - ΔSI = ΔNSI.
Saeterbakken et al. [22]	♀ office workers with neck or shoulder pain ≥ 2 and ≥ 3 m *n* = 34 ♀ (end 31 ♀) **SI group** *n* = 13 (end 12) 47.6 ± 11.9 y **NSI group** *n* = 10 (end 9) 41.0 ± 15.3 y **REF group** *n* = 11 (end 10) 50.3 ± 14.8 y	**5 neck-shoulder specific strengthening ex with elastic bands** - 30′ supervised training 2 times/w for 10 w - ≥2 d between sessions - 3 × 12 reps (3″/rep) - 1′ pause between ex. - Loads that allowed 12 reps, ending at or near to fatigue. When 17 reps, load progressively ↑	**Nordic walking** - 30′ supervised training 2 times/w for 10 w - ≥ 2 d between sessions - Moderate intensity. Progressively ↑ (Borg 6–20 scale) - Nordic walking poles: individually adjusted REF group - No physical act	**Pain:** - Pain intensity (last 5 days’ mean) (VAS1) ~ baseline ~ post-intervention (10 w) ~ 10 w follow-up	**Short-term effects (post-intervention):** VAS 1: - ↓ SI and NSI; = REF - ΔSI = ΔNSI = ΔREF **Long-term effects (follow-up):** VAS1: - ↓ SI? (*p* = 0.058) and NSI; =REF - ΔSI = ΔNSI = ΔREF

Abbreviations: ′, minute/s; ″, second/s; ↑, increased/higher; ↓, decreased/lower; =, no change; ♂, male subjects; ♀, female subjects; ACT, activity/ies; D, day/s; EX, exercise/s; ITT, intention to treat analysis; M, month/s; n, number of subjects; NRS, numerical rating scale; NSI, non-specific intervention; PD, pain drawings; REF, reference group; REP/S, repetition/s; RM, repetition maximum; SI, specific intervention; VAS, visual analogical scale; W, week/s; Y, years.

**Table 4:** Study characteristics. Specific strength vs. body-mind exercises.

Reference	Characteristics of Participants	Specific Intervention (SI)	Non-Specific Intervention (NSI) + Reference Intervention (REF)	Outcome Measures / Follow-Up Period	Main Results
Ahlgren et al. [28]	♀ < 45 y with trapezius myalgia for ≥1 y + sick leave ≤ 1 m last y *n* = 136 ♀ (−34 excluded/withdrew = 102 ♀) 38.2 y **SI1 group** *n* = 29 ♀ 38.0 ± 6.0 y **SI2 group** *n* = 28 ♀ 38.5 ± 5.6 y **NSI group** *n* = 25 ♀ 37.6 ± 6.1 y **REF group** *n* = 20 ♀ 38.9 ± 5.4 y	- 3 × 1 h/w supervised for 10 w - 15′ general warm-up - Last 10′: stretching **SI1 group: 4 neck-shoulder specific strengthening concentric ex with air machines** - Load individualized to 2 × 12 RM - Load ↑ when 3 sets = comfortable **SI2 group: endurance training with arm ergometer alternated with specific arm ex. with rubber expanders** - 4 × 3′ arm ergometer (110–120 bpm) - Specific arm ex: 3′ - Expanders individually loaded to allow 30–35 RM/ex/set (3 sets)	**Body awareness** - 3 × 1 h/w supervised for 10 w - 15′ general warm-up - Muscular tension awareness and relaxation - Attention focused on balance, posture and breathing **REF group** - 1 × 2 h/w supervised for 10 w - No physical act. Learn and discuss stress management	**Pain:** - Pain at present (VAS1) - Pain in general (VAS2) - Pain at worst (VAS3) ~ baseline ~ post-intervention (10 w) **PPTs**: 6 trigger points in the 3 portions of the trapezius (TP) muscle (TP2, TP4, TP5), 2 sides (R, L) **PD:** Pain distribution and pain character (% total body area)	**Short-term effects (post-intervention):** VAS1: - ↓ SI1, SI2 and NSI; = REFVAS2: - ↓ SI1, SI2 and NSI; = REFVAS3: - ↓ SI1, SI2 and NSI; = REF- ΔSI1 & ΔSI2 > ΔREF
Waling et al. [29]					**Short-term effects (post-intervention):** VAS 1: - ↓ SI1, SI2, NSI; = REF - ΔSI1 = ΔSI2 = ΔNSI = ΔREF - Δ“exercisers” (SI1 + SI2 + NSI) > ΔREF VAS 2: - ↓ SI1, SI2, NSI; = REF - ΔSI1 = Δ SI2 = ΔNSI = ΔREF VAS3: - ↓ SI1, SI2, NSI; = REF - ΔSI1 and ΔSI2 > ΔREF - Δ“exercisers” (SI1 + SI2 + NSI) > ΔREF PPTs: - ↑SI2 (in 2 trigger points); = SI1, NSI and REF - TP2L: ΔSI1 < ΔSI2 ΔSI2 > ΔREF - TP5L: ΔSI2 > ΔREF ΔNSI > ΔREF - TP2R, TP5R and TP5L: Δ“exercisers” (SI1 + SI2 + NSI) > ΔREF PD: - ΔSI1 = ΔSI2 = ΔNSI = ΔREF
Cramer et al. [30]	Patients (18–60 y) + non-specific neck pain VAS ≥ 4 and ≥ 3 m *n* = 51 42 ♀ and 9 ♂ 47.8 ± 10.4 y VAS 4.5 ± 1.9 **SI group** *n* = 26 21 ♀ and 5 ♂ 49.5 ± 9.5 y **NSI group** *n* = 25 21 ♀ and 4 ♂ 46.2 ± 11.2 y	**Specific neck**–**shoulder posture awareness, stretching and strengthening ex** - 10′/d (home ex) for 9 w - Self-care manual - Sitting position - Use of a towel as an aid - Diary	**Yoga** - 90′ yoga session/w for 9 w: - 10–15 patients - 8–10 yoga postures/session - Last 15′ relaxation - Iyengar yoga type - 3 sitting + 3 standing postures - No previous experience in yoga - + 10′/d (home ex) - Diary	**Pain:** - Pain at rest (VAS1) - Pain at motion (VAS2) (after 6 reps of head flex, ext, lateral flex R/L, rotation R/L) (mean pain intensity of the 6 movements) **SF36-BP** (bodily pain items) **PPTs:** - Maximal pain site (PPT) ~ baseline ~ post-intervention (9 w)	**Short-term effects (post-intervention):** VAS1: - ↓NSI; = SI - ΔNSI > ΔSI VAS2: - ↓ SI & NSI - ΔSI = ΔNSI SF36-BP: - ↑ NSI; = SI - ΔNSI > ΔSI PPTs: - ↑ NSI; = SI - ΔNSI > ΔSI
Viljanen et al. [31]	♀ office workers (30–60 y) with chronic non-specific neck pain ≥ 3 m *n* = 393 ♀ (end 340 ♀) **SI group** *n* = 135 ♀ (end 111 ♀) 45 ± 6.6 y **NSI group** *n* = 128 ♀ (end 110 ♀) 43 ± 7.3 y **REF group** *n* = 130 ♀ (end 119 ♀) 44 ± 7.4 y ITT analysis	**Specific neck**–**shoulder dynamic strengthening ex with dumbbells** - 3 times/w for 12 w + reinforcement training for 1 w - Supervised groups ≤ 10 people - 1–3 kg according to RM test with 7.5 kg - Intensity progressively ↑ - Stretching after each ex	**Relaxation** - 3 times/w for 12 w + reinforcement training for 1 w: - Supervised groups ≤ 10 people - Progressive relaxation method, autogenic training, functional relaxation and systematic desensitisation - ≠Techniques being incorporated through the 12 w **REF group** - Ordinary act - No supervision	**Pain:** - Pain intensity (VAS) ~ Baseline ~ post-intervention (13 w) ~ 3 m follow-up ~ 9 m follow-up	**Short-term effects (post-intervention):** VAS: - =SI, NSI and REF - ΔSI = ΔNSI = ΔREF **Long-term effects (both follow-up moments):** VAS: - =SI, NSI and REF - ΔSI = ΔNSI = ΔREF

Abbreviations: ′, minute/s; ↑, increased/higher; ↓, decreased/lower; =, no change; ≠, different; ♂, male subjects; ♀, female subjects; CI, confidence interval; D, day/s; EX, exercise/s; EXT, extension; FLEX, flexion; ITT, intention to treat analysis; KG, kilogram/s; L, left; M, month/s; n, number of subjects; NSI, Non-specific intervention; PD, pain drawings; R, right; REF, reference group; REPS, repetitions; RM repetition maximum; SI, specific intervention; VAS, visual analogical scale; W, week/s; Y, years.

**Table 5:** Study characteristics. Specific stretch vs. general stretch.

Reference	Characteristics of Participants	Specific Intervention (SI)	Non-Specific Intervention (NSI) + Reference Intervention (REF)	Outcome Measures / Follow-Up Period	Main Results
Cunha et al. [27]	♀ (35–60 y) with chronic neck pain lasting ≥ 3 m *n* = 33 ♀ (end 31 ♀) **SI group** *n* = 17 (end 16) 48.7 ± 7.3 y **NSI group** *n* = 16 (end 15) 44.4 ± 7.8 y	**Static neck-shoulder stretching ex** - 60′ × 2/w for 6 w - 30′ manual therapy and breathing ex + 30′ conventional auto-passive stretching - 2 × 30″/ex	**Global posture reeducation stretching** - 60′ × 2/w for 6 w - 30′ manual therapy and breathing ex + 30′ supervised muscle chain stretching - 2 postures: posterior and anterior chains (15′/posture)	**Pain:** - Pain intensity (VAS) **SF36-BP** (bodily pain items) ̴ baseline ̴ post-intervention (6 w) ̴ 6 w follow-up	**Short-term effects (post-intervention):** VAS: - ↓ SI and NSI - ΔSI = ΔNSI SF36-BP: - ↑ SI and NSI - ΔSI = ΔNSI **Long-term effects (follow-up):** VAS: - ↓ SI and NSI - ΔSI = ΔNSI SF36-BP: - ↑ SI and NSI - ΔSI = ΔNSI

Abbreviations: ′, minute/s; ″, second/s; ↑, increased/higher; ↓, decreased/lower; =, no change; ♀, female subjects; EX, exercise; M, month/s; n, number of subjects; NSI, Non-specific intervention; REF, reference group; SI, specific intervention; VAS, visual analogical scale; W, week/s; Y, year/s.

## Data Availability

The data presented in this study are available from the corresponding author upon reasonable request.
